# Recovery and characterisation of quinoa protein via neutral to alkaline extraction: protein profiles and heat-induced aggregation behaviour

**DOI:** 10.1016/j.crfs.2025.101214

**Published:** 2025-09-30

**Authors:** Hao Cui, Debashree Roy, Siqi Li, Trevor S. Loo, Qing Guo, Aiqian Ye

**Affiliations:** a Riddet Institute, Massey University, Private Bag 11 222, Palmerston North, 4442, New Zealand; b School of Food Technology and Natural Sciences, Massey University, Private Bag 11 222, Palmerston North, 4442, New Zealand; c School of Fundamental Sciences, Massey University, Palmerston North, 4474, New Zealand; d College of Food Science and Nutritional Engineering, China Agricultural University, Beijing, 100083, China

**Keywords:** Quinoa protein, Protein composition, Alkaline extraction, Aggregation, Functionality

## Abstract

This study investigated the impact of extraction pH (pH 7–11) on the yield, composition, protein profile and properties of quinoa protein isolate (QPI), including solubility, interfacial tension and heat-induced aggregation. Extraction pH considerably influenced protein yield, purity and solubility, with alkaline conditions promoting higher protein yield (from 27.4 % to 39.5 % as extraction pH increased from pH 7 to pH 11) but lower purity (from 89.7 % to 85.1 %) owing to the co-precipitation of carbohydrates. Sodium dodecyl sulphate–polyacrylamide gel electrophoresis analysis combined with liquid chromatography with tandem mass spectrometry revealed major protein profile in quinoa protein, confirming the abundance of 11S globulin. Moreover, extraction pH affected the solubility over a broad range of pH, interfacial properties and heat-induced aggregation behaviour of QPI. QPI extracted at pH 9 exhibited better solubility and better interfacial tension reduction capability but lower heat stability compared with QPI extracted at pH 11. Results from this study provided useful information and essential insights into the utilisation of quinoa proteins in various food applications.

## Highlights

• Extreme alkaline enhanced co-precipitation of carbohydrates.
• Mild alkaline extraction improved solubility and interfacial properties of QPI.
• Extreme alkaline extraction improved heat stability of QPI.
• 7S and 11S globulin participated in the heat-induced aggregation of QPI.

## Graphical abstract

**Figure:**
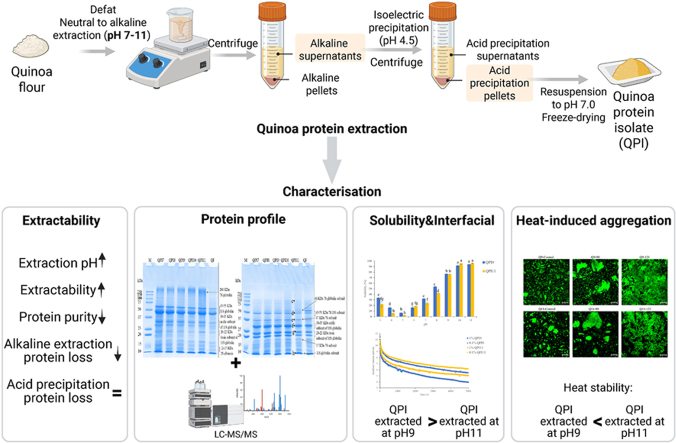

## 1 Introduction

In recent years, quinoa (Chenopodium quinoa Willd.) has garnered considerable attention as a promising source of plant-based proteins owing to its balanced amino acid profile and excellent nutritional properties. Notably, essential amino acids were present in quinoa protein in proportions closely aligned with human nutritional needs (FAO, 2011). The essential amino acid content of quinoa was higher than of another pseudocereal, amaranth (Lopez et al., 2018). Compared with other plant proteins, the lysine content of quinoa protein was comparable to that of soybean and exceeds that of wheat, maize and rice (Abugoch James, 2009; Okon, 2021). Its gluten-free nature, agronomic characteristics and nutritional superiority rendered quinoa highly versatile for various food applications (Cui et al., 2023).

It was established that the seed storage proteins of quinoa mainly comprised 11S globulins (∼37 %) and 2S albumins (∼35 %) (Dakhili et al., 2019). The 11S globulins consisted of small basic and larger acidic polypeptides linked by a disulfide bonds, forming a hexamer structure (Dakhili et al., 2019). 11S globulins comprised small basic and larger acidic polypeptides linked by a disulphide bonds, forming a hexamer structure (Dakhili et al., 2019). The 2S albumin in quinoa protein formed a heterodimer structure from polypeptides of ∼8–9 kDa linked by disulphide bonds (Brinegar et al., 1996). Apart from 11S globulins and 2S albumins, recent studies identified legumin-like 13S globulins and vicilin-like 7S globulins in quinoa seeds via proteomics analysis (Burrieza et al., 2019; Shen et al., 2022; Van de Vondel et al., 2020). The 13S and 7S globulins of quinoa have not been fully characterised; however, similar proteins were found in other pseudocereals (Campos Assumpção de Amarante et al., 2024; Van de Vondel et al., 2020). The 13S globulin of quinoa resembled the 11S globulin of buckwheat in structure, whereas the 7S globulin of amaranth was in a tetrameric form of ∼200 kDa with sub-units of 16, 38, 52 and 66 kDa (Janssen et al., 2017; Quiroga et al., 2010).

The extraction method significantly influenced the characteristics and functional properties of quinoa protein. Commonly extracted via alkaline extraction–isoelectric point precipitation method, quinoa protein isolate (QPI) exhibited varied purity and functional properties based on extraction pH (Liu et al., 2023; Ruiz et al., 2016b; Van de Vondel et al., 2020). Higher alkaline extraction pH led to decreased purity of protein isolates and protein denaturation and subsequently affected protein aggregation and functional properties (Cui et al., 2023). For instance, QPI extracted at pH 10–11 could be used in liquid foods such as sauces and soups, whereas that extracted at pH 8–9 could be employed in protein beverages or gelled foods, as the latter extracts demonstrated improved thermal and gelling properties (Abugoch et al., 2008; Campos Assumpção de Amarante et al., 2024). Structural changes in QPI occurred with increasing extraction pH, impacting solubility and heat-induced gel strength (Liu et al., 2023). To date, a few studies investigated the functional properties of QPIs extracted from various alkaline pH, mainly focusing on extractability, solubility, structure and gelation properties (Abugoch et al., 2008; Elsohaimy et al., 2015; Liu et al., 2023; Ruiz et al., 2016a, 2016b). The protein profile and heat-induced aggregation behaviour of QPI from various extraction pHs remained unclear, requiring a detailed investigation to tailor the production of QPI with desired functionality.

In this study, QPI was extracted under varying pH (pH 7–11), followed by isoelectric precipitation at pH 4.5 and subsequent freeze drying. First, protein yield and purity were compared among different extraction pHs as well as protein composition obtained from alkaline extraction supernatants (AES), alkaline extraction pellets (AEP) and resulting QPI as revealed by sodium dodecyl sulphate–polyacrylamide gel electrophoresis (SDS–PAGE). The protein profile of QPI was further revealed through in-gel digestion and liquid chromatography with tandem mass spectrometry (LC–MS/MS) based on NCBI quinoa database. Moreover, functional properties, including solubility, interfacial tension and heat-induced aggregation of QPI extracted at pH 9 and 11, were investigated. This study aimed to elucidate how extraction pH affected the protein composition and functional properties of QPI. The insights gained from the results of this study will aid in selecting the optimal extraction pH for desired properties, thereby enhancing the utility of QPI in food formulation.

## 2 Materials and methods

### 2.1 Materials

Whole grain quinoa flour was purchased from Kiwi Quinoa Inc. (Taihape, New Zealand). All chemicals were obtained from Sigma Chemical Co. (St. Louis, MO, USA) unless otherwise specified. Milli-Q water (water purified by treatment with a Milli-Q apparatus, Millipore Corp., Bedford, MA, USA) was used for the preparation of all solutions.

### 2.2 Preparation of QPI

**Fig. 1:**
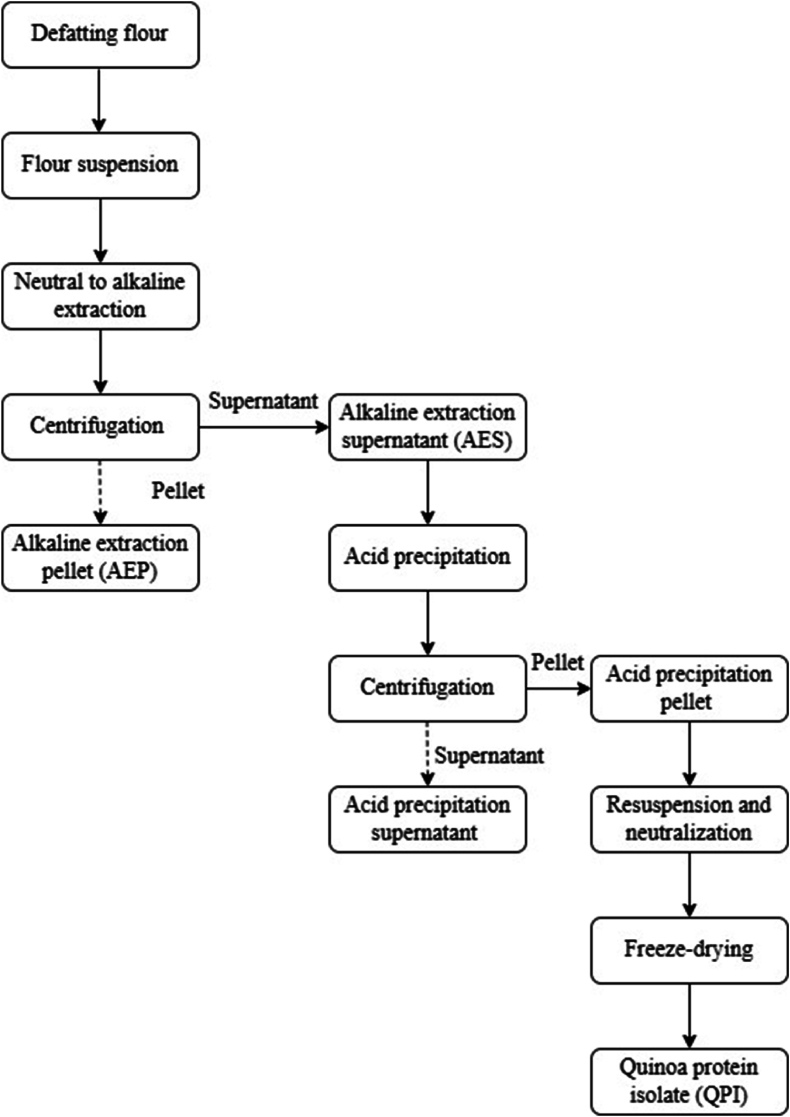
Preparation of quinoa protein isolates (QPI). Solid line indicates retained samples. Dotted line indicates discarded samples.

QPI was prepared via alkaline solubilisation‒isoelectric precipitation according to Liu et al. (2023) with slight modifications, as shown in Fig. 1. Briefly, quinoa flour was defatted using hexane at a ratio of 1:3 (flour/hexane (w/v)) for 1 h with continuous stirring at room temperature (25 °C). This defatting procedure was repeated three times. The defatted flour was then placed in a fume hood overnight at room temperature to remove the hexane residue.

The defatted flour was suspended in water (10 % w/w) with pH adjusted to 7, 8, 9, 10 and 11. These suspensions were stirred at 300 rpm for 90 min at room temperature and then centrifuged at 9000 *× g* and 25 °C for 15 min. After centrifugation, the supernatants were precipitated by adjusting pH to 4.5 with 6 M HCl, followed by centrifugation of suspensions at 9000 *× g* and 25 °C for 15 min. QPI was obtained by resuspending the pellets in Milli-Q water and neutralising the solution using 6 M NaOH to pH 7 and subsequent freeze drying. The freeze-dried isolates were stored at −20 °C for further analysis.

### 2.3 Determination of protein yield, protein purity and protein loss

The protein content of samples was determined using the Kjeldahl method with a conversion factor of 6.25 (Elsohaimy et al., 2015). Protein yield, purity, and loss were calculated as follows using Eqs. (1), (2), (3) (Ruiz et al., 2016a, 2016b):

(1) $$Protein\;yield\;\left(\%\right)=\frac{isolate\;protein\;content\;\left(\%\right)\times isolate\;weight\;\left(g\right)}{flour\;protein\;content\;\left(\%\right)\times flour\;weight\;\left(g\right)}\times 100\%$$

(2) Proteinpurity(%)=isolateproteincontent(%)

(3) $$Protein\;loss\;\left(\%\right)=\frac{protein\;weight\;at\;start\;of\;each\;step\;\left(g\right)-protein\;weight\;after\;each\;step\;\left(g\right)}{flour\;protein\;content\;\left(\%\right)\times flour\;weight\;\left(g\right)}\times 100\%$$

### 2.4 Chemical composition of QPI

Sample moisture was determined according to AOAC International (1995) that heating of samples was conducted in an oven at 105 °C for 3 h. Fat content was determined according to AACC 30-10 (AACC, 1983), by acid hydrolysis of the sample with HCl followed by extraction of hydrolysed lipid materials with mixed ethers. Ash was determined by gravimetry difference of incinerated sample in muffle at 550 °C (AOAC International, 1995). Carbohydrate content was calculated by difference (FAO, 2003).

### 2.5 Visualization of protein composition

The protein composition of alkaline extraction supernatants (AES), alkaline extraction pellets (AEP) and resulting quinoa protein isolates (QPI) was determined using SDS–PAGE under non-reducing and reducing conditions in a Mini PROTEIN II system (Bio-Rad Laboratories, Richmond, CA, USA). The concentration of resolving and stacking gels was 16 % and 4 %, respectively. All samples were mixed with either a sample buffer [0.625 mM Tris-HCl buffer, pH 6.8, 10 % (v/v) glycerol, 2 % (w/v) SDS, 0.04 % (w/v) bromophenol blue] (non-reducing) or sample buffer containing 5 % (v/v) β-mercaptoethanol (reducing) to reach a protein content of 4 mg/mL. In addition, reducing samples were boiled for 10 min, and all samples were centrifuged at 12,000 rpm for 2 min at 25 °C before loading to resolving gels. Each well contains 5 μL of samples. An electrophoresis analysis was conducted on a constant voltage of 120 V for ∼90 min until the bromophenol blue dye line reached the bottom of the gel. Gels were stained for 1 h with a Coomassie Brilliant Blue R-250 solution and then destained with a destaining solution overnight. Subsequently, gels were visualised using a Bio-Rad Molecular Imager Gel Doc XR + imaging system. Densitometric analysis of gels was conducted using the Bio-Rad Image Lab software version 6.1. A protein marker (Precision Plus Protein™ Standards) (Catalogue # 1610374, Bio-Rad, USA) with a molecular weight range of 10–250 kDa was loaded to SDS-PAGE gel together with the samples.

### 2.6 Liquid chromatography tandem mass spectrometry (LC-MS/MS)

Selective SDS–PAGE gel protein bands were identified through in-gel digestion followed by LC–MS/MS analysis. The digestion of bands was carried out using trypsin according to the protocol described by Shevchenko et al. (2006) with some modifications. Briefly, gel bands from colloidal Coomassie-stained SDS–PAGE gels were cut into small pieces, placed in protein LoBind tubes (Eppendorf, Germany) and destained with 50 % acetonitrile in 50 mM ammonium bicarbonate at 45 °C. The gel pieces were dehydrated with 500 μL of 50 % acetonitrile and dried under vacuum centrifugation for 10 min (Savant SpeedVac, Thermo Fisher Scientific, USA). Approximately 30 μL of 0.15 % (v/v) dithiothreitol (Sigma-Aldrich, USA) in LC–MS-grade water (Fisher Scientific, Belgium) was added and incubated at 45 °C for 1 h to reduce the disulphide bonds in dried gel fragments. The supernatant was then removed, and the gel pieces were rinsed with 100 μL of 50 mM ammonium bicarbonate. Any remaining liquid of ammonium bicarbonate was removed, and gel pieces were dehydrated with 300 μL of 80 % acetonitrile again, followed by drying in a SpeedVac. For alkylation, the gel pieces were incubated with 30 μL of 0.37 % (w/v) iodoacetamide in ammonium bicarbonate for 30 min in the dark, followed by rinsing with LC–MS-grade water, dehydrating with 80 % acetonitrile twice and thorough drying in the SpeedVac for 10 min. The gel pieces were rehydrated with 130 μL of 20 ng/μL trypsin in 50 mM ammonium bicarbonate and rested on ice for 10 min. Excess solution of trypsin and ammonium bicarbonate mixture was removed and replaced by 50 mM ammonium bicarbonate for incubation at 37 °C overnight. The next day, the gel pieces were sonicated (Elmasonic, Elma, Switzerland) for 2 min, and the supernatant was collected in a new LoBind tube. The sonication of gel pieces and collection of supernatant was repeated with 110 μL of 5 % (v/v) formic acid (Thermo Fisher Scientific, USA) in 40 % (v/v) acetonitrile and again with 0.1 % (v/v) formic acid in 80 % (v/v) acetonitrile. The combined supernatants were reduced to 30 μL in a SpeedVac and stored in 2-mL vials with a glass micro insert (Thermo Fisher Scientific, USA) at −80 °C prior to LC–MS analysis.

Peptide separation in the digests was performed using high-performance liquid chromatography (HPLC) on a Dionex UltiMate™ 3000 RSLC nano system (Thermo Fisher Scientific, USA), which incorporates an online reversed-phase peptide trap (PepMap100 C18, 3 μm particle size, 75 μm ID, 2 cm length) and a reverse-phase capillary analytical column (PepMap100 C18, 2 μm particle size, 75 μm ID, 50 cm length; Thermo Fisher Scientific, USA). The HPLC system was interfaced with a Q Exactive™ Plus Hybrid Quadrupole-Orbitrap™ MS, featuring a higher-energy collision-induced dissociation collision cell, an Orbitrap™ mass analyser and a Nanospray Flex™ Ion Source (Thermo Fisher Scientific, USA). The gradient ranged from 3 % to 35 % acetonitrile in 0.1 % formic acid water over 60 min, with a flow rate of 300 nL/min. Eluted peptides were subjected to data-dependent tandem MS acquisition (top 10) methodologies, performing a survey scan at a resolution of 70,000 within the mass range of 375–1600 m/z. The top 10 most intense peptide ions underwent fragmentation at a resolution of 17,500 for the acquisition of sequence information. This scanning process was iteratively conducted throughout the chromatographic run, incorporating settings to exclude repetitive ions within specified time windows (6 s), thus enhancing the targeting of low abundance ions and overall coverage. The ionisation source operated at a voltage of 1.5 kV, with a capillary temperature of 250 °C, an S-Lens RF level of 50 %, an isolation width of 1.4 m/z and a normalised collision energy of 28.

The raw data files were analysed using the Proteome Discoverer™ search engine (version 2.4.1.15, Thermo Fisher Scientific, USA). The search parameters aligned with the instrument specifications of Q Exactive Plus. The variables from chemical treatments and potential natural modifications of proteins are listed in Table 1. Identification confidence was calculated by Percolator using a reverse decoy database, and all protein hits were filtered to meet a false discovery rate of 1 % or better and include at least two unique peptides.

**Table 1:** Data analysis parameter.

Search engine	Proteome Discoverer v 2.4.1.15
Databases	*Chenopodium quinoa* from NCBI on May 16, 2023 + contaminant databases: cRAP, from The Global Proteome Machine, and CCP cRAP from Cambridge Centre for Proteomics
Enzyme	No enzyme (non-specific cleavage)
Max # of missed cleavages	2
Min peptide length	6
Precursor mass tolerance	10 ppm
Fragment mass tolerance	0.02 Da
Static modifications	Carbamidomethyl (C)
Variable modifications	Peptides: Oxidation (M), Deamination (N/Q). Protein N-terminal: Acetylation, Met-loss, Met-loss + acetylation.
False Discovery Rate (FDR)	1 % or less
Display filter	# of unique peptides ≥2

### 2.7 Determination of protein solubility

QPI extracted at pH 9 (QPI9) and 11 (QPI11) were selected for the following characterisations, because QPI9 maintained some structural integrity while QPI11 was fully denatured in a previous study (Abugoch et al., 2008). The pH of 1 % (w/w) QPI suspensions was adjusted to 3–11 using 6 M HCl or 6 M NaOH with continuous stirring for 1 h. The suspensions were centrifuged at 10,000 rpm (17880 g) for 15 min at 25 °C to divide them into supernatants and pellets. The protein content of supernatants and QPI was measured using the Kjeldahl method with a conversion factor of 6.25. The protein solubility was calculated using Eq. (4):

(4) $$Solubility\;\left(\%\right)=\frac{supernatant\;protein\;content\;\left(\%\right)\times supernantant\;weight\;\left(g\right)}{isolate\;protein\;content\;\left(\%\right)\times isolate\;weight\;\left(g\right)}\times 100\%$$

### 2.8 Determination of interfacial tension

The time evolution of the oil–water interfacial tension of QPI suspensions (1 % and 0.1 %, pH 7) was measured using the Theta Flex Plus optical tensiometer (Biolin Scientific Instruments, Västra Frölunda, Sweden) and calculated through automation by the One Attention software (Biolin Scientific Instruments, Västra Frölunda, Sweden) using the pedant drop method according to Luo et al. (2022) with slight modifications. A QPI suspension droplet with a constant volume (15 μL) was immersed into 2 mL corn oil in a disposal cuvette using a stainless-steel syringe tip. The evolution of interfacial tension was monitored for 5000 s.

### 2.9 Characterisation of the heat-induced protein aggregation

#### 2.9.1 Sample preparation

QPI9 and QPI11 were dissolved in water, followed by adjusting the suspension pH to 7 with 6 M NaOH. Protein concentrations for both suspensions were 3.5 % based on the purity of isolates to mimic common plant-based beverage systems. Suspensions were stirred overnight at room temperature for complete hydration. An aliquot of 3-mL suspensions was subject to heating either at 90 °C for 10 min using a water bath or at 121 °C for 10 min using an oil bath. Unheated suspensions from each extraction pH were considered as control. Once heat treatment was completed, the samples were cooled down to room temperature using ice bath. In the following sections, the samples were labelled as follows: Q9-control (QPI extracted at pH 9 without heat treatment), Q9-90 (QPI extracted at pH 9 and treated at 90 °C for 10 min), Q9-121 (QPI extracted at pH 9 and treated at 121 °C for 10 min), Q11-control (QPI extracted at pH 11 without heat treatment), Q11-90 (QPI extracted at pH 11 and treated at 90 °C for 10 min) and Q11-121 (QPI extracted at pH 11 and treated at 121 °C for 10 min).

#### 2.9.2 Characterisation of particle size

The particle size distributions of samples were determined in triplicate by static light scattering using a Mastersizer 2000 laser diffraction particle size analyser (Malvern Instruments Ltd., Worcestershire, UK) integrated with an automated small-volume liquid dispersion unit (Hydro, 2000S). The refractive indices of 1.45 and 1.33 were assigned to the proteins and the continuous phase (water), respectively. The particle size was calculated as the volume-weighted average diameter (d_4,3_, μm) using Eq. (5):

(5) $$d_{4,3}=\sum \frac{n_{i}d_{i}^{4}}{n_{i}d_{i}^{3}}$$

where $n^{i}$ denotes the number of particles with diameter of $d^{i}$.

#### 2.9.3 Determination of soluble protein content

The soluble protein content of control and heat-treated samples was determined according to the method of Bogahawaththa et al. (2019) as an indication of heat stability. Heated and unheated samples were centrifuged at 700 g for 10 min at 25 °C to mimic the natural deposition of sediments that commonly occur in protein beverages. Protein content was determined using the Kjeldahl method with a conversion factor of 6.25. Soluble protein content was calculated using Eq. (6)

(6) $$Soluble\;protein\;\left(\%\right)=\frac{Protein\;content\;of\;heated\;or\;unheated\;supernatatnt}{Protein\;content\;of\;the\;original\;dispersion}\times 100\%$$

#### 2.9.4 Microstructure of control and heat-treated samples

The microstructure of samples was visualised using a Leica TCS SP5 confocal laser scanning microscope (Leica Microsystems, Wetzlar, Germany) fitted with a 40 × oil immersion objective at room temperature. Samples were mixed with fast green FCF (1 mg/mL in water) with a ratio of 50:1 (V_sample_/V_dye_) to stain proteins. Stained samples were transferred onto concave microscopy slides and covered with a glass coverslip. Representative confocal graphs were captured and processed using Zeiss ZEN 3.1 (blue edition) imaging software (Carl Zeiss, Germany).

### 2.10 Statistical analysis

The results were expressed as the calculated means ± standard deviations. QPI extraction was carried out in two independent batches, and each physicochemical measurement from each batch was performed in at least duplicate. Statistical analysis was performed using Minitab software (Minitab version 19, Minitab Inc., State College, PA) to determine the significance of the differences. At a 95 % confidence level (*P* < 0.05), one-way analysis of variance and Tukey's pairwise comparison test were used in the statistical analysis.

## 3 Results and discussion

### 3.1 Impact of extraction pH on extractability and chemical composition

**Fig. 2:**
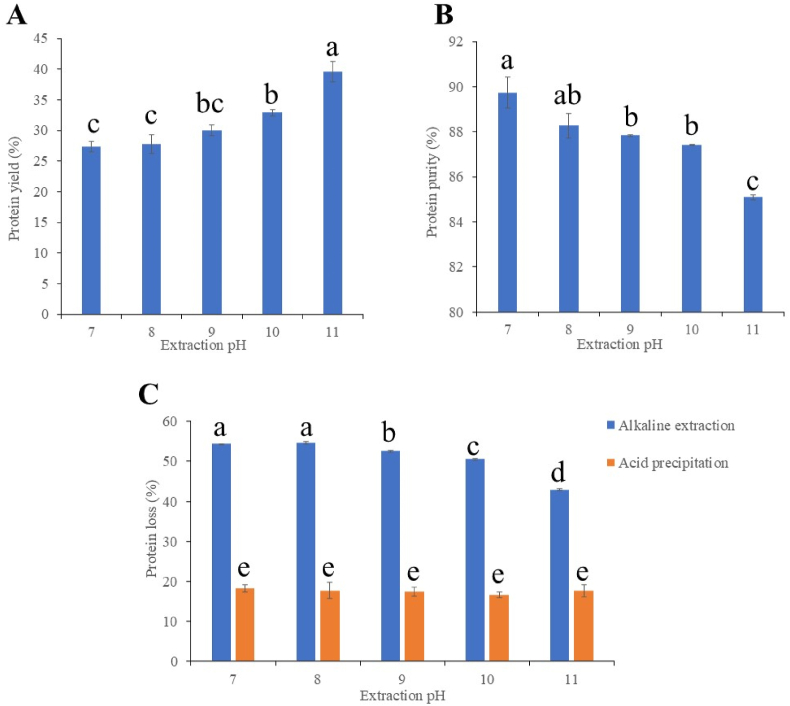
(A) Protein yield and (B) protein purity of quinoa protein isolates extracted at different pHs. (C) Protein loss during alkaline extraction and acid precipitation at different extraction pHs. Bars with different letters are significantly different at p < 0.05.

The protein yield exhibited an increasing trend, i.e. from 27.4 % ± 0.9 %–39.5 % ± 1.7 % as extraction pH increased from pH 7 to pH 11 (Fig. 2A). However, protein purity slightly decreased from 89.7 % ± 0.7 % at pH 7–85.1 % ± 0.1 % at pH 11 as extraction pH increased (Fig. 2B). This phenomenon was in line with previous results from Liu et al. (2023) and Ruiz et al. (2016b). Increasing extraction pH significantly increased the carbohydrate content of QPI, but did not affect the fat, moisture and ash content (Table 2). When extraction pH increases from neutral (pH 7) to high alkaline (pH 8 or higher), the protein solubility increased due to the deprotonation of the amine groups, ionisation of the carboxyl groups and disruption of the disulphide bonds under high alkaline conditions (Liu et al., 2023), which in turn induced the elevated protein yield. Nevertheless, an extreme high alkaline pH (higher than pH 9) also led to the co-precipitation of non-protein components (e.g. fibre and starch) with the protein isolates into extraction suspensions, thereby explaining the reduction in protein purity (Lestari et al., 2010).

**Table 2:** Chemical composition of QPI extracted under different pHs.

Extraction pH	Fat (%)	Moisture (%)	Ash (%)	Carbohydrate (%)
7	1.22 ± 0.01^a^	3.54 ± 0.03^a^	4.81 ± 0.08^a^	0.70 ± 0.59^a^
8	1.27 ± 0.04^a^	3.75 ± 0.16^a^	4.79 ± 0.13^a^	1.93 ± 0.21^b^
9	1.18 ± 0.02^a^	3.67 ± 0.05^a^	4.68 ± 0.04^a^	2.64 ± 0.03^b^
10	1.28 ± 0.02^a^	3.55 ± 0.08^a^	4.75 ± 0.04^a^	3.30 ± 0.12^b^
11	1.28 ± 0.06^a^	3.66 ± 0.09^a^	4.70 ± 0.03^a^	5.28 ± 0.24^c^

The majority of protein loss occurred during alkaline extraction (Fig. 2C), that is, protein loss showed a decreasing trend as extraction pH increased from pH 7 to pH 11. No significant difference in protein loss was observed during acid precipitation. The protein loss during alkaline extraction concurred with the outcomes of the increase in protein yield as the extraction pH increased. This indicated that more protein was solubilised from the grain matrix, ultimately contributing to the composition of the final protein isolates (Ruiz et al., 2016b).

### 3.2 Protein profiles

**Fig. 3:**
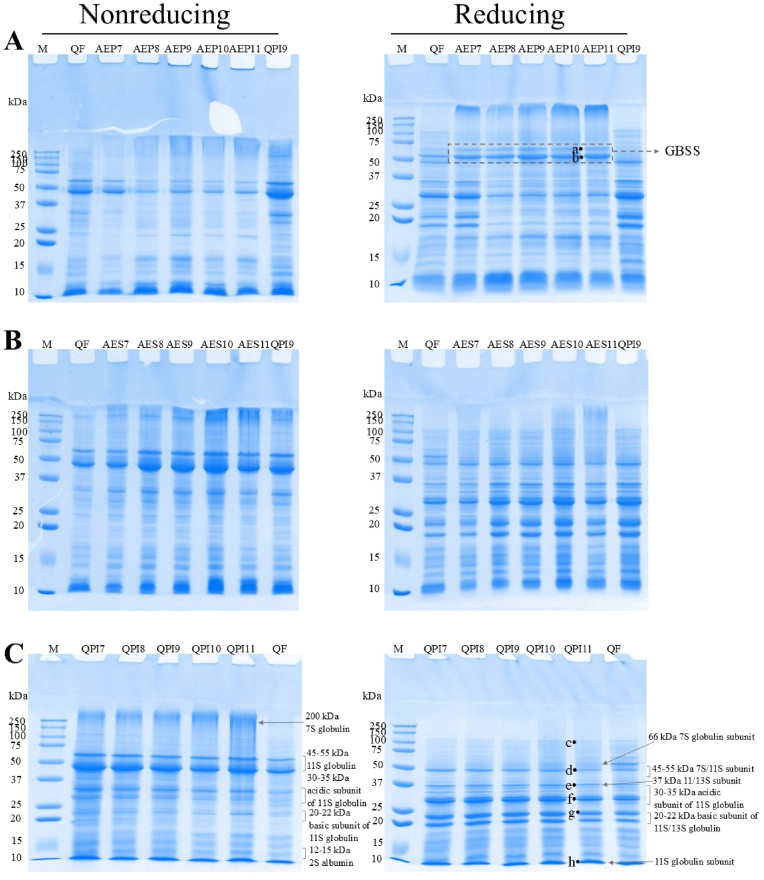
SDS-PAGE protein profiles of (A) alkaline extraction pellet (AEP), (B) alkaline extraction supernatant (AES), and (C) quinoa protein isolate (QPI), at different extraction pH (pH 7–11) under nonreducing and reducing conditions. GBSS, granule-bound starch synthases. M, marker. QPI9, quinoa protein isolate extracted at pH 9. QF, quinoa flour. 7–11, extraction pH.

The composition of alkaline extraction pellet (AEP), alkaline extraction supernatant (AES) and quinoa protein isolate (QPI) at different extraction pHs (pH 7–11) was analysed using SDS–PAGE under non-reducing and reducing conditions (Fig. 3). As shown in Fig. 3A, AEP samples showed two protein bands between 50 kDa and 75 kDa without a disulphide linkage (bands **a** and **b**), identified by LC–MS/MS as granule-bound starch synthases with molecular masses of 62 and 56 kDa, located within the central core of the starch grains (Table 3), and were removed from alkaline extraction (Lindeboom et al., 2005; López-Fernández et al., 2021). Additionally, AEP samples displayed protein bands over 75 kDa and below 37 kDa at all extraction pHs, indicating protein loss primarily during alkaline extraction (Fig. 2C). AES and QPI exhibited similar protein compositions under both non-reducing and reducing conditions (Fig. 3B and C). The quinoa seed storage protein 11S globulin was reduced to acidic (30–35 kDa) and basic sub-units (20–22 kDa), indicating the occurrence of disulphide bonds in 11S globulin. Protein bands identified via LC–MS/MS are summarised in Table 3. The protein band **c** with a molecular weight of ∼100 kDa contained mainly enzymes that regulate plant growth. The protein band **d** remained intact under reducing conditions, suggesting that it could be a non-covalently bonded sub-unit of 7S vicilin-like globulins or unreduced 11S globulin (Burrieza et al., 2019; Liu et al., 2023). Protein bands **e**, **f** and **g** were confirmed to be sub-units of 11S and 13S globulins, with bands **e** and **f** as acidic sub-units and band **g** as a basic sub-unit of 11S globulin (Abugoch et al., 2008; Luo et al., 2022). The protein band **h**, with a molecular weight of 10 kDa, was identified as a sub-unit of 11S globulin and not of the commonly considered 2S albumin (Brinegar et al., 1996; Campos Assumpção de Amarante et al., 2024; Luo et al., 2022). Similar results were also reported by Van de Vondel et al. (2020) that they identified a group of SDS-PAGE protein bands with apparent molecular weights between 6 and 17 kDa to be basic subunits of legumin-like proteins and subunits of vicilin-like proteins instead of 2S albumin. Globulins of many sources had an isoelectric point between pH 4 and 5, whereas albumins were soluble over a wide pH range (pH 2–9) (Gruppen and Voragen, 2005). Albumins remained soluble and were discarded during acid precipitation; consequently, fractions from the conventional alkaline extraction–acid precipitation method mainly contained globulins (Yang et al., 2024). This was consistent with a similar protein loss during acid precipitation across pH 7–11, as shown in Fig. 2C, indicating that the extraction pH did not change the loss of albumin during acid precipitation. LC–MS/MS analysis confirmed the abundance of 11S globulin in QPI.

**Table 3:** Accession number, protein description, molecular weight, the amount of peptides^a^ and unique peptides^b^ identified, and coverage^c^ of proteins identified with mass spectrometry from bands (a-g) of the sodium dodecyl sulphate–polyacrylamide gel electrophoresis (SDS-PAGE) under reducing conditions in Fig. 3.

SDS-PAGE band	Accession number	Protein description	Molecular weight (kDa)	Peptides	Unique peptides	Coverage (%)
a	ALB36799.1	GBSSIb [Chenopodium quinoa]	66.9	32	13	66
b	ALB36788.1	GBSSIa [Chenopodium quinoa]	66.9	32	13	68
c	XP_021745077.1	heat shock cognate protein 80 [Chenopodium quinoa]	79.8	32	25	55
	XP_021715208.1	5-methyltetrahydropteroyltriglutamate--homocysteine methyltransferase [Chenopodium quinoa]	85.4	24	18	52
	XP_021726756.1	alpha-glucosidase-like [Chenopodium quinoa]	101.1	22	11	43
	XP_021774521.1	elongation factor 2-like [Chenopodium quinoa]	93.6	24	24	47
	XP_021766485.1	pullulanase 1, chloroplastic-like [Chenopodium quinoa]	106.1	22	5	46
d	XP_021762713.1	vicilin-like antimicrobial peptides 2-1 [Chenopodium quinoa]	63.9	21	13	41
	XP_021768838.1	11S globulin seed storage protein 2-like [Chenopodium quinoa]	52.4	13	13	55
e	XP_021752668.1	13S globulin seed storage protein 2-like [Chenopodium quinoa]	61	24	24	53
	XP_021768838.1	11S globulin seed storage protein 2-like [Chenopodium quinoa]	52.4	11	11	44
f	AAS67036.1	11S seed storage globulin [Chenopodium quinoa]	53.6	19	8	46
	ABI94736.1	11S seed storage globulin B [Chenopodium quinoa]	53.5	20	9	52
	XP_021768838.1	11S globulin seed storage protein 2-like [Chenopodium quinoa]	52.4	16	16	53
g	XP_021752668.1	13S globulin seed storage protein 2-like [Chenopodium quinoa]	61	20	20	44
	XP_021768838.1	11S globulin seed storage protein 2-like [Chenopodium quinoa]	52.4	22	9	61
h	AAS67036.1	11S seed storage globulin [Chenopodium quinoa]	53.6	9	2	33

a The number of peptides per protein have been detected.

b The amount of the detected peptide sequences unique to a protein group.

c The percentage of protein sequence supported by detected tryptic peptides.

### 3.3 Impact of extraction pH on the solubility of quinoa protein isolate

**Fig. 4:**
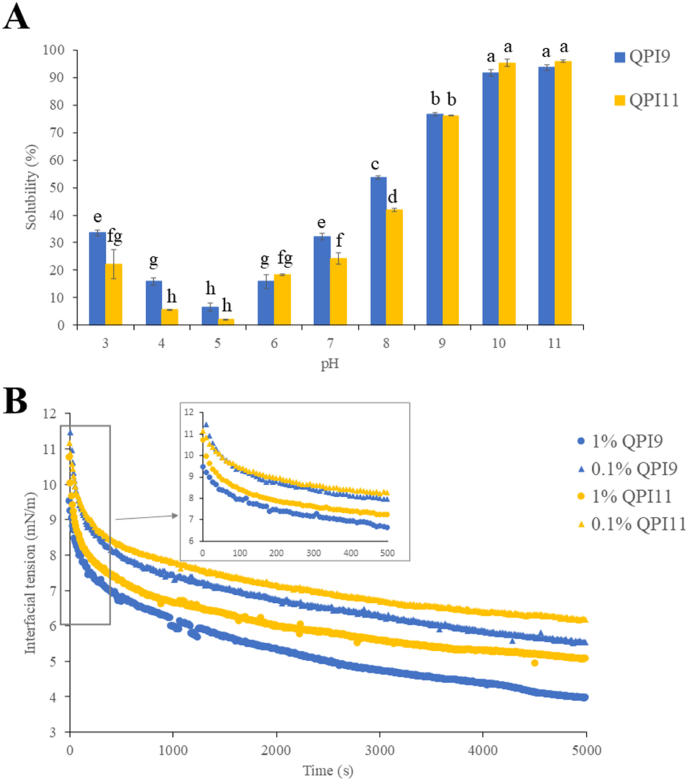
(A) Solubility of QPI9 and QPI11 at pH 3 to pH 11. (B) Interfacial tensions of quinoa protein suspension at various concentrations from various extraction pHs. QPI9, quinoa protein isolate extracted at pH9. QPI11, quinoa protein isolate extracted at pH11.

The solubility of QPI was lowest at pH 4–5 but increased at pH 6 onwards (Fig. 4A), consistent with the solubility of QPI reported by Ruiz et al. (2016b), Elsohaimy et al. (2015) and Mäkinen et al. (2015). Elsohaimy et al. (2015) reported that the solubility of QPI exhibited a U-shaped dependence on pH (in the pH range of 1–10), being relatively low (25 %) at pH 4 and reaching a maximum (75 %) at ∼ pH 10. The lowest solubility at pH 4–5 may be attributed to 11S globulin of quinoa, the major protein in QPI, whose isoelectric point is ∼pH 4.5–5.0 (Shen et al., 2021). This U-shaped solubility profile, with the lowest solubility at the isoelectric point, is similar to most legume proteins (Grossmann and McClements, 2023). QPI9 exhibited a significantly higher solubility than QPI11 at pH 3–4 and pH 7–8. Ruiz et al. (2016b) reported similar results, in which QPI extracted at pH 8 and 9 exhibited higher solubility than QPI extracted at pH 10 and 11. Additionally, amaranth proteins isolated at pH 9 showed a significantly higher solubility than those isolated at pH 10–12 across pH 3–9 (Das et al., 2021). This could be explained by the exposure of hydrophobic groups led by the denaturation of proteins at extreme alkaline conditions (Das et al., 2021; Ruiz et al., 2016b). Liu et al. (2023) reported a significant increase in particle size and a decrease in surface charge for QPI when extraction increased from pH 9 to pH 11, indicating that extreme alkaline extraction caused severe protein denaturation and molecule aggregations. Moreover, extreme alkaline conditions induce the exposure of hydrophobic groups and decrease surface polarity, leading to insoluble protein aggregations via hydrophobic interactions (Abugoch et al., 2008; Ruiz et al., 2016b). Gao et al. (2020) also reported that pea proteins isolated at pH 9.5 exhibited a lower solubility at both neutral and acidic environment pHs than those extracted at pH 8.5; the protein aggregation in isolates from higher extraction pH was confirmed by the increased total sulfhydryl group amount and decreased exposed free sulfhydryl group amount. Moreover, the composition (i.e. the presence of non-protein components, such as starch, dietary fibres, minerals, and lipids) also affected the solubility (Grossmann and McClements, 2023). QPI11 contained more non-protein components than QPI9 (Fig. 2B). Phenolic compounds in QPI were also associated with the reduction in solubility, especially at low pH, through non-covalent and covalent bonding with quinoa protein (Giteru et al., 2023).

### 3.4 Impact of extraction pH on the interfacial properties of quinoa protein isolate

All QPI suspensions showed a similar behaviour, in which the interfacial tension initially dropped significantly over time and eventually reached an equilibrium at the end of the measurement (5000 s) (Fig. 4B). QPI suspensions with a lower protein concentration (0.1 %) displayed higher initial interfacial tension than those with a higher protein concentration (1 %). Similar results were reported by Zhang et al. (2021), in which the initial interfacial tension decreased from around 15.5 mN/m to 12.2 mN/m when the concentration of QPI suspensions increased from 1 % to 5 %. Proteins typically migrate from the aqueous phase to the oil–water interface, where they adsorb and adopt a stable conformation over time (Tang, 2020). It was found that extraction pH strongly affected the interfacial tension reduction for both concentrations, with QPI9 demonstrating a higher interfacial tension reduction capacity compared with QPI11. Generally, higher protein solubility enhances the ability to reduce interfacial tension, as proteins need to be dissolved first before they can migrate to the interface (Karaca et al., 2011). This was consistent with the higher solubility of QPI9 than that of QPI11 at pH 7 (Fig. 4A). In addition, QPI9 had highly hydrophobic unfolded polypeptides with a better efficiency in absorbing and stabilising the interface, leading to its higher surface hydrophobicity than QPI11 (Liu et al., 2023), which could partially explain the better interfacial tension reduction capability for QPI9 (Mitropoulos et al., 2014; Mota da Silva et al., 2021). Interfacial properties are essential because they are highly related to the emulsifying properties of proteins. QPI9 demonstrated better interfacial properties, suggesting that it is more suitable for utilisation in emulsion systems than QPI11.

### 3.5 Impact of extraction pH on the heat-induced aggregation of QPI

**Fig. 5:**
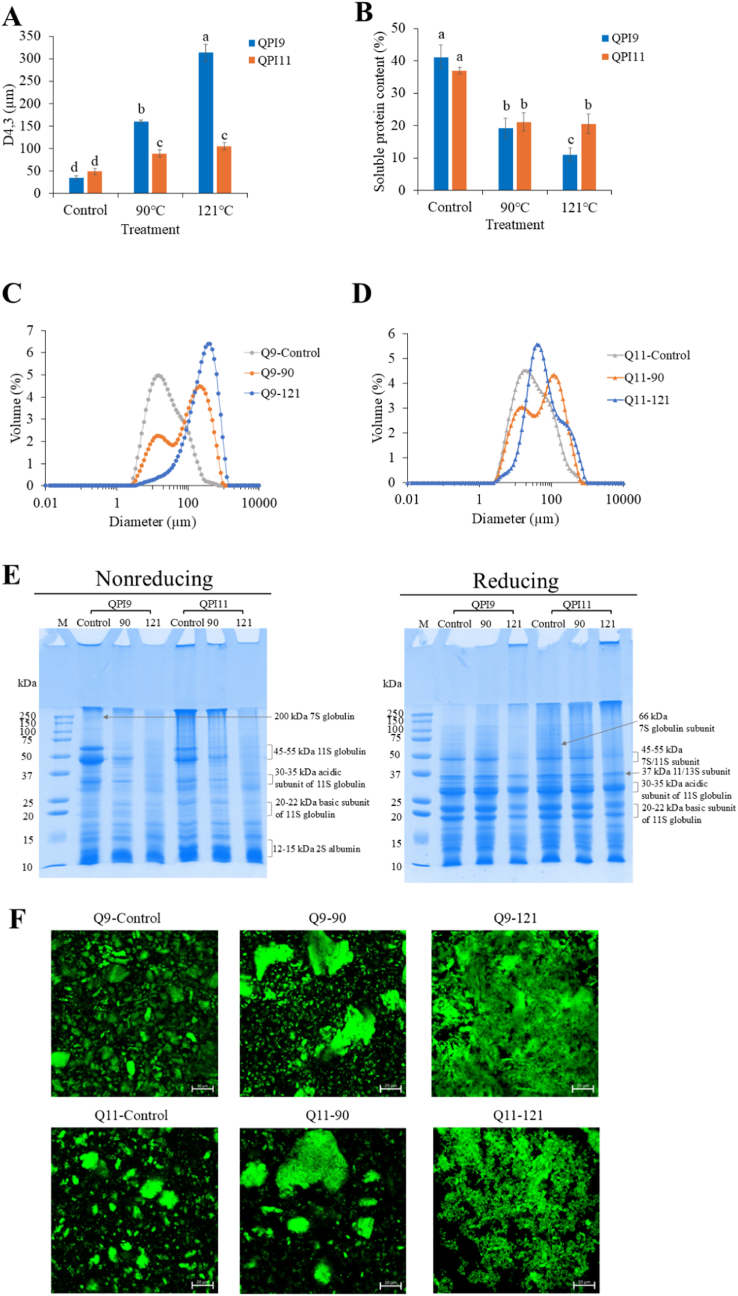
(A) Particle sizes of control and heat-treated quinoa protein isolate (QPI). (B) Solubility of control and heat-treated QPI samples. (C) Size distributions of control and heat-treated QPI9 samples. (D) Size distributions of control and heat-treated QPI11 samples. (E) Protein profiles of control and heat-treated QPI samples under nonreducing and reducing conditions. (F) Microstructures of control and heated QPI samples under CLSM. Scale bar indicates 20 μm. QPI9, QPI extracted at pH9. QPI11, QPI extracted at pH11.

Heat treatments significantly increased the particle size of QPI and decreased the solubility for both extraction pHs (Fig. 5A and B). Size distribution further supported this trend that control samples showed a narrow peak at smaller particle sizes, whereas heat-treated samples exhibited broader, bimodal distributions with a clear shift toward larger aggregates, especially in QPI9 (Fig. 5C and D). The decrease in solubility of heat-treated samples confirmed the formation of insoluble aggregates. Similar results were reported for pea protein isolates (Bogahawaththa et al., 2019) and hemp protein isolates (Raikos et al., 2015). Plant proteins usually unfold and expose non-polar groups when they are subjected to heating above 50 °C, resulting in protein aggregation and precipitation through hydrophobic and covalent interactions (Bogahawaththa et al., 2019). As solubility decreased, particle size increased, indicating a strong correlation between protein aggregation and reduced solubility. QPI11 showed a lesser extent of increase in particle size and decrease in solubility compared with QPI9 (Fig. 5A and B). As a result, QPI11 showed a slightly better heat stability than QPI9. Ruiz et al. (2016b) also revealed that QPI extracted at pH 9 had a significantly larger particle size when it was subjected to heating at 60°C–90 °C than QPI extracted at pH 11. They suggested that QPI extracted at higher pH may have a greater degree of denaturation, which could hinder further association and aggregation during heat treatment, whereas QPI extracted at lower pH with a lesser degree of denaturation still had the functional capacity for aggregation (Ruiz et al., 2016b).

The protein profiles of control and heated QPI suspensions under non-reducing and reducing conditions are displayed in Fig. 5E. Under non-reducing conditions, the intensity of the bands was progressively reduced as the heating temperature increased for both QPI9 and QPI11 compared with controls, indicating the formation of non-soluble aggregates that were unable to be separated into the gel. For control samples, 7S globulin at 200 kDa, 11S globulin as well as its acidic and basic sub-units and 2S albumin can be identified easily. When QPI9 and QPI11 were heated at 90 °C for 10 min, 11S globulin (45–55 kDa) mainly participated in the formation of large aggregates based on the decreased band intensity, while its sub-units remained visible in the SDS–PAGE lanes. When the heating temperature increased to 121 °C for 10 min, 7S globulin was further involved in the aggregation. The result suggests that intact 11S globulin (45–55 kDa) was more prone to covalently aggregate when subjected to heating compared with its acidic and basic sub-units (Van de Vondel et al., 2021). Additionally, 2S albumin did not form non-soluble aggregates. Van de Vondel et al. (2021) reported that when the heating temperature reached 100 °C, quinoa 7S and 11S globulins formed aggregates predominantly via disulphide bonds but 2S albumins did not participate in covalent protein aggregation.

Under reducing conditions, samples heated at 90 °C for 10 min showed identical protein profiles as controls for both QPIs. However, samples heated at 121 °C for 10 min showed a reduced band intensity for 7S and 11S globulins. This suggested that covalent interactions other than disulphide bonds may also contribute to the formation of non-soluble aggregates when samples were subjected to heating at 121 °C (Raikos et al., 2015). However, QPI9 and QPI11 did not show different protein profiles for heat-induced aggregation.

The microstructure of heated QPI samples differed between QPI9 and QPI11, especially when subjected to heating at 121 °C (Fig. 5F). In the control samples, QPI9 displayed slightly smaller particles compared with QPI11. Control samples displayed an average particle size of 35.08 μm and 46.84 μm for QPI9 and QPI11, respectively, similar to the particle size of freeze-dried QPI (44.24 μm) as reported by Shen et al. (2021). Previous studies suggested that alkaline extraction followed by isoelectric precipitation promoted the formation of aggregates through hydrophobic interactions between globulins, with the extent of aggregation influenced by processing procedures (Luo et al., 2022). Both QPI9 and QPI11 exhibited heat-induced aggregation following heating at 90 °C. Big protein clumps (>100 μm) and non-aggregated small particles (<20 μm) were unevenly distributed, consistent with the bimodal size distribution. When the heating temperature increased to 121 °C, the heterogeneous interconnected protein network that was present in both QPIs and QPI9 appeared to form a denser protein matrix than QPI11. These varied microstructures were attributed to differences in the intrinsic protein structures between QPI9 and QPI11. Proteins extracted at mild alkaline pH (as in QPI9) were known to retain more globular structures, which could aggregate more densely upon heating due to stronger hydrophobic interactions and disulphide bonding (Liu et al., 2023; Ruiz et al., 2016b). Conversely, QPI11 underwent greater protein denaturation during extraction, leading to the flocculation and sedimentation of larger particles while inhibiting the aggregation of smaller particles, thereby reducing the tendency to form a strong network (Ruiz et al., 2016b; Wang et al., 2024).

## 4 Conclusions

This study demonstrated the considerable influence of extraction pH on the protein profile and the functionality of QPI. Alkaline extraction conditions enhanced protein yield but decreased protein purity due to the co-precipitation of carbohydrates. SDS–PAGE combined with LC–MS/MS revealed the protein profile and confirmed the abundance of 11S globulin as a seed storage protein. Additionally, extraction pH affected the solubility, interfacial properties and heat-induced aggregation of QPI. The reduced solubility of QPI11 at certain pH levels compared with QPI9 may result from a higher degree of protein aggregation and the presence of more non-protein components. QPI9 showed a stronger capacity for interfacial tension reduction than QPI11 due to its better solubility and the presence of highly hydrophobic unfolded polypeptides. Heat treatment led to significant aggregation in QPI, with both 7S and 11S globulins forming non-soluble aggregates as temperature increased, especially during heating at 121 °C. Moreover, heat treatments led to the increase in particle size and decrease in solubility for both QPI9 and QPI11, as well as varied microstructures, although QPI11 demonstrated better heat stability. The varied functionality can be exploited for the development of specific food applications. For instance, QPI extracted at mild alkaline pH (pH 9) appears more suitable for emulsified food applications, such as dressings and sauces, while QPI extracted at extreme alkaline pH (pH 11) may be more appropriate in thermally processed foods, such as protein beverages. While interfacial tension results suggest potential emulsifying functionality, emulsifying properties were not evaluated in this study. Therefore, future work should include direct emulsifying characterisation to establish the practical relevance of QPI in emulsion-based systems. In conclusion, extraction pH needs to be considered for incorporating QPI into food formulation for desired properties.

## CRediT authorship contribution statement

Hao Cui: Writing – original draft, Methodology, Investigation, Formal analysis, Visualization. Debashree Roy: Writing – review & editing, Supervision. Siqi Li: Writing – review & editing, Supervision. Trevor S. Loo: Writing – review & editing, Software, Methodology, Formal analysis. Qing Guo: Writing – review & editing, Supervision. Aiqian Ye: Conceptualization, Funding acquisition, Resources, Project administration, Writing – review & editing, Supervision.

## Declaration of competing interest

The authors declare that they have no known competing financial interests or personal relationships that could have appeared to influence the work reported in this paper.
